# A study on the relationship between mindfulness and work performance of web editors: Based on the chain mediating effect of workplace spirituality and digital competencies

**DOI:** 10.3389/fpsyg.2022.1068735

**Published:** 2023-01-18

**Authors:** Jiazi He, Xinwei Li, Huiyi Wang, Zhiwu Xu

**Affiliations:** ^1^The Editorial Department of Periodicals, Guangdong University of Petrochemical Technology, Maoming, China; ^2^Faculty of International Tourism and Management, City University of Macau, Macao, Macao SAR, China; ^3^The School of Transmedia, Guangzhou Academy of Fine Arts, Guangzhou, China; ^4^Faculty of Humanities and Social Sciences, City University of Macau, Macao, Macao SAR, China; ^5^School of Information Technology in Education, South China Normal University, Guangzhou, China

**Keywords:** mindfulness, work performance, workplace spirituality, digital competencies, web editors

## Abstract

**Introduction:**

Based on the job demands-resources model, this study aims to explore the relationship between mindfulness in web editors, work performance, workplace spirituality, and digital competencies.

**Methods:**

Online data from the Tencent Questionnaire Platform was used to examine the proposed research model. We distributed questionnaires to new media companies, and a total of 431 valid questionnaires were collected.

**Results and Discussion:**

The results suggested that mindfulness in web editors can improve workplace spirituality, digital competencies, and work performance. In addition, workplace spirituality was found to act as a mediator between mindfulness and work performance. And, digital competencies did not play a mediating role between mindfulness and work performance, but workplace spirituality and digital competencies played a chain mediating role between mindfulness and work performance. The study explained the internal impact mechanism of mindfulness on work performance in web editors, and proposed methods to improve mindfulness, revealing the chain mediating role of workplace spirituality and digital competencies in the impact of mindfulness on work performance, which might provide new insights into existing research. It can provide a reference for new media companies to manage the team of web editors and improve the work performance of web editors.

## Introduction

Today, we live in an era of Omnimedia. According to the 49th Statistical Report on Internet Development in China as of December 2021, the number of netizens in China has reached 1.032 billion, and the Internet penetration rate has reached 73.0%. The new media industry is developing rapidly, and web editors play an important role as employees of new media companies. Web editors are an emerging profession with the rapid development of the Internet. Their main job content is to use their own professional knowledge and computer software and hardware knowledge and other modern information technologies to design and build website content (Wu, 2016). Therefore, web editors are defined as “someone who rely on the Internet, engage in the production and dissemination of online information and knowledge in units or institutions with online publisher qualifications, and provide online publications and related services to online user groups to achieve social and economic benefits.” Wu (2016) stated that, different from traditional media editors, web editors have the following characteristics, namely mastering network editing operation skills, flexible real-time interaction capabilities, and editing integration capabilities for resource libraries. This puts forward higher requirements for the work competencies of web editors. Most of the existing research on web editors focused on the cultivation of web editors talents and the improvement of their quality (Mu, 2010), but had not specifically research the working environment of web editors, nor explore how to improve the work performance of web editors from the perspective of psychology. This study fills the research gap in this field.

Since the COVID-19 outbreak in 2020, web editors have exerted strong initiative and dynamism to strengthen the production and dissemination of high-quality network information knowledge content. As an important part of the editorial team, web editors are an important support for the development of media integration in China (Wang, 2022). However, at the same time, a small number of online publishers, driven by economic interests, have excessive pursuit of click-through rate, ranking list, and update rate, and have affected the career of web editors’ value and job identity by adjusting the platform’s internal operating system, assessment and incentive mechanisms (Wang, 2022). Compared to employees in other organizations, web editors face greater career challenges and occupational pressures. Therefore, the professional ethics and professional quality of the web editors team need to be strengthened, and how to effectively improve the work competencies (e.g., digital competencies) and work performance of web editors has become a serious issue for new media companies. Early research has noted that the modern workplace is often full of distractions that challenge attentional control, impair occupational functioning, and even affect performance (Jett and George, 2003). Web editors face greater concentration challenges than other professional employees. It is particularly important to effectively improve the mindfulness of web editors. And, it is necessary to improve the stability of attention through mindfulness practice and help web editors focus on the current work (Hasenkamp et al., 2012), thereby improving work performance.

Currently, there is growing enthusiasm for mindfulness research, and mindful-based training programs are gaining popularity in the workplace to reduce stress, improve employee well-being and boost output. With organizations, including Google, Aetna, Mayo Clinic, and the U.S. Army used mindfulness training to enhance workplace productivity (Tan et al., 2012; Wolever et al., 2012; West et al., 2014; Jha et al., 2015). After mindfulness training, people see life stress not as difficulty or disaster, but as modifiable ways to relieve emotional stress (Liu et al., 2022a). In addition, mindfulness enables employees to accept a variety of information, breaks fixed thinking, and is more likely to have conflicts and brainstorming, which is conducive to employees generating innovative ideas (Chen et al., 2022a). In fact, since the 1980s, mindfulness—a Buddhist spiritual practice, has been researched in the field of psychology (Bishop et al., 2004), and has been defined by some scholars as present-centred attention and awareness (Brown and Ryan, 2003). Mindfulness was being used in multiple fields, with research in disciplines such as psychology, medicine, and neuroscience. The research provided that mindfulness affects attention, cognition, emotion, behavior, and physiology in positive ways (Good et al., 2016). Organizational sociology and organizational psychology scholars have also shown great enthusiasm in the concept of mindfulness (Reb and Atkins, 2015; Good et al., 2016), and have gradually applied mindfulness training to organizational practice.

Existing research on mindfulness has established that mindfulness can improve work performance, reduce turnover intentions, etc. (Dane and Brummel, 2014), help employees understand themselves and others, and improve work engagement (Federman, 2009; Kahn, 2010). The association between mindfulness and workplace spirituality has been studied by previous study (Petchsawang and McLean, 2017), mindfulness and competencies (Liu et al., 2022b), however few research have examined the connection between workplace spirituality and digital competencies in mindfulness and work performance. Although the existing research has extended the competencies to digital competencies, it mainly focuses on the digital competencies of teachers in China to meet the needs of teachers to be competent for digital education in the future. This study fills the research gap on digital competencies for web editors and analyzes the further work and literacy requirements for web editors in the Internet era, and studies the relationship between digital competencies, work performance, mindfulness, and workplace spirituality. Therefore, in order to reveal the relationship between mindfulness and web editors’ workplace spirituality, digital competencies and work performance, this study mainly applies the job requirement-resource (JD-R) model and resource conservation theory, attempts to examine mindfulness as a cognitive resource how to improve workplace spirituality, digital competencies, and work performance of web editors.

The main purposes of this study are: (1) to explore whether mindfulness has a significant positive impact on the workplace spirituality, digital competencies, and work performance of web editors; whether the workplace spirituality has a positive effect on the digital competencies and work performance of web editors and whether digital competencies has a significant positive impact on the work performance of web editors; (2) to explore whether workplace spirituality and digital competencies have a mediating role between web editors’ mindfulness and work performance; (3) to explore whether there is a chain mediating role in the relationship between mindfulness and work performance of workplace spirituality and digital competencies. Theoretically, this is the first study to investigate the impact mechanism between mindfulness and workplace spirituality, digital competencies and work performance. In this regard, the comprehensive model presented in this study extends research on mindfulness and work performance while making theoretical contributions to the current management literature. In practice, the results of this study can provide useful suggestions for the improvement of work performance of web editors, that is, by improving the mindfulness of web editors can affect their workplace spirituality and digital competencies, and ultimately can promote their work performance, thereby promoting development of new media companies.

## Theoretical basis and hypothesis development

### Theoretical basis

The job demand-resource (JD-R) model, based on Karasek’s (1979) job-demand control model and Siegrist's (1996, 2002) effort-reward imbalance model, was introduced as an alternative to the employees health and well-being. It originally proposed by Demerouti et al. (2001), and was initially only applied to job burnout. A few years later, Schaufeli and Bakker (2004) incorporated engagement into the JD-R model, and Xanthopoulou et al. (2009) incorporated personal resources into the model as well. These three papers have been cited tens of thousands of times in Google Scholar, indicating that the JD-R model has received extensive attention in the academic and practical circles, and has achieved a lot of research results. The core assumption of the JD-R model is that although each occupation may have factors related to working conditions, these conditions can be divided into two broad categories of job demands and job resources, thus forming an overall model. Bakker and Demerouti (2007) published the article The Resource Model for Job Requirements: Recent Developments, which made an in-depth analysis of the related concepts of the JD-R model and outlined the roadmap of the JD-R model.

According to literature review, JD-R model has two different roadmaps. The first one is from Bakker and Demerouti in 2007, they said, the health-damaging process and the motivation-driven process are two relatively distinct but interdependent causal processes found in the JD-R model. The health-damaging process is where high job demands lead to negative organizational outcomes by increasing stress; the motivation-driven process is where job resources may act as intrinsic motivators, due to the fact that they foster employees’ development, learning, and growth and may also play an extrinsic motivating function, by satisfying the basic needs of occupational individuals (Deci and Ryan, 1985), promoting their formation of motivation that helps to achieve work goals, thereby positively affecting positive organizational outcomes.

Another roadmap is from Schaufeli in 2017, he describes the JD-R model roadmap somewhat differently from Bakker and Demerouti (2007). The roadmap clearly marked the path of health-damaging process and the motivation-driven process, the mediator variable was burnout or work engagement, and the dependent variable was negative outcome or positive outcome. Compared with Bakker and Demerouti (2007) using the outcome of the organization as the dependent variable, it is equivalent to expanding the scope of the dependent variable, and the negative or positive impact on the individual is also included in the scope of the outcome variable. Furthermore, the JD-R model generalized by Bakker and Demerouti (2007) shows an interaction effect between job demands and resources, but Schaufeli (2017) argues that current evidence on demand-resource interaction effects suggests that, even if significant, this actual correlation of these interactions is also very low (Xanthopoulou et al., 2007), therefore, the two causal processes included in the JD-R model are essentially independent, and many existing empirical studies are based on one of the path of the JD-R model to design research models (Wang et al., 2021; Xu and Lin, 2021). The JD-R model can be said to be an influential framework for understanding how job demands or job resources foster employee well-being (Lesener et al., 2019).

### The relationship between the JD-R model and this study

**Figure 1:**
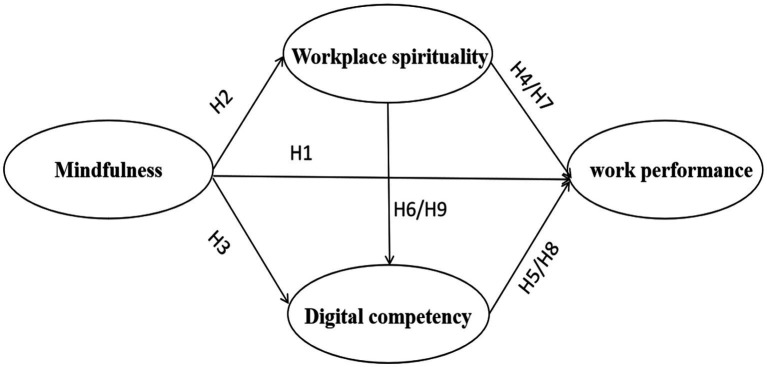
Hypothesis model.

Based on the motivation-driven process of the JD-R model, this study designs a research model to improve job performance by reducing job burnout caused by job requirements by providing job resources. Job resources are components of the work that might expedite task completion, reduce the psychological cost of job requirements, and enhance work performance (Huang et al., 2022). In this study, the independent variable mindfulness is the psychological resource of web editors, which corresponds to work resources and helps web editors to focus on the current work, reduce negative emotions at work, and improve work performance. Job demands are different aspects of job conditions that call for a consistent amount of physical or mental effort (Huang et al., 2022). In this study, the mediating variables workplace spirituality and digital competencies are the performance of a kind of work ability generated by job resources motivated by occupational individuals, which require continuous efforts to cultivate individuals. Finally, the dependent variable work performance corresponds to positive outcomes for the organization. Therefore, the research logic of this study is as follows: mindfulness, as an individual psychological resource for web editors, can not only play an internal motivating role, positively affect workplace spirituality, and then positively affect work performance; it can also play an external motivating role to promote employees improve digital competencies, which in turn positively affects the work performance of web editors. At the same time, because there is a correlation between workplace spirituality and competencies, it is assumed that workplace spirituality and digital competencies play a chain mediating role between mindfulness and work performance. Accordingly, the model of this study is constructed based on the model of the JD-R model, as shown in Figure 1.

## Hypothesis development

### Mindfulness and work performance

Due to empirical studies demonstrating the benefits of mindfulness on one’s psychological, physical, and performance, it has recently drawn more attention (Hyland et al., 2015). Initially, mindfulness was thought to be a fundamental aspect of the Buddhist tradition and was mostly explored in the context of philosophy and religious studies (Gunaratana, 2001). In 1993, scholars brought mindfulness to the management literature, but they reframed it as an emphasis on novelty and cognitive flexibility (Weick and Roberts, 1993), which was largely different from the Buddhist concept. Receptive attention and awareness of what is going on right now are two characteristics of mindfulness (Brown and Ryan, 2003; Quaglia et al., 2015). Being mindful involves being consciously alert, in the present, and without passing judgement on anything (Liu et al., 2022c). Its core features, receptive attention to current events, reflected in a persistent awareness of ongoing experience (Brown and Ryan, 2003), demonstrate the importance of mindfulness in web editors’ work. Instead of being a concept, mindfulness is described as a set of skills, that is, it includes the ability to: (1) observe and pay attention to numerous stimuli; (2) concentrate and take action; (3) respond to non-evaluative verbal descriptions of observations; (4) avoid immediate evaluation (Baer et al., 2006).

Collective attention is referred to as mindfulness at the organisational level, and it is said that practising mindfulness helps leaders and staff make fewer mistakes, stay alert, and respond to unforeseen situations in a productive way (Rerup and Levinthal, 2014). Work performance refers to how successfully employees perform their duties and contribute to the outcomes and success of the business (Shooshtarian et al., 2013). Research has shown that mindfulness can affect the functioning of teams and organizations, and numerous previous studies have shown a direct relationship between employee mindfulness and work performance (Shao and Skarlicki, 2009). Regarding the indirect relationship between mindfulness and work performance, scholars Ngo et al. (2020) proved through empirical research that creative process participation and employee creativity mediate the relationship between mindfulness and work performance of service employees, which is a new breakthroughs in research of the indirect influence mechanism of mindfulness. In addition, studies have shown that mindfulness practice is effective in increasing work engagement and indirectly affects work performance through its impact on work engagement (Huang et al., 2022).

According to Baytos and Kleiner (1995), it is possible to measure work performance accurately using a variety of factors, including job quality, punctuality, performance, productivity, etc. The three qualities of attention that mindfulness can influence positively are stability, control, and efficiency (Good et al., 2016). Mindfulness prevents mind wandering by maintaining focus in the present. Mindfulness as a personal resource can help individuals maintain a state of concentration at all times, avoid possible distractions in the workplace, and increase productivity (Huang et al., 2022). According to resource conservation theory, all individuals have a tendency to retain and accumulate valuable resources, which in organizational settings may include working conditions, job rewards, social support, employment opportunities, and personal energy (Hobfoll, 1989; Dollard and Bakker, 2010), while mindfulness is able to improve the quality of attention and enable individuals to harness these available energy resources to produce more effective creative work (Montani et al., 2018). As a result, mindful individuals are able to direct their attention to related needs rather than distraction, they are better able to direct their attention to their tasks with greater stability, control and efficiency, resulting in expanding their effective attention skills. Therefore, they might be able to comprehend information more effectively and act more logically as a result (Kirk et al., 2011), thereby guaranteeing the quality of work by reducing individual errors due to inattention, while at the same time controlling and stabilizing the current task by effectively controlling and stabilizing attention to information to help individuals demonstrate better task performance (Smallwood and Schooler, 2015). From this, we assume that:

***Hypothesis 1***: Mindfulness has a significant positive effect on work performance.

### Mindfulness and workplace spirituality

Purity of mind is the foundation of healthy living (Pawar, 2008). In fact, a person with a pure heart can acquire the human values of health and wholesomeness, which are the basis of workplace spirituality. In other words, workplace spirituality requires purification of the mind and heart (Petchsawang and McLean, 2017). Workplace spirituality is distinct from religion, which involves formally structured institutional belief systems, while spirituality focuses on personal experience from inner strength (Petchsawang, 2008). Workplace spirituality refers to “having compassion for others and experiencing a conscious inner awareness in pursuit of meaningful work, thus achieving transcendence” (Petchsawang and Duchon, 2009). At the same time, workplace spirituality can deeply understand the organizational context in which employees need “mental enrichment, spiritual fulfillment, soul satisfaction and financial reward” (Dhiman and Marques, 2011). Liu et al. (2022d) provided a deeper understanding of animated pedagogical-guided loving-kindness meditation by linking mindfulness, spirituality, and subjective well-being and indicated that mindfulness has a positive effect on subjective well-being. And, high levels of mindfulness may allow for high spiritual experiences, which would increase a person’s subjective well-being (Liu et al., 2020a). Mindfulness, on the other hand, is a “state of being awake and attentive, not mixed with competing images, thoughts, or feelings” (Heaton et al., 2004), which helps people identify their needs, problems, and conflicts (Brown and Ryan, 2003), resulting in effective emotion regulation, increased empathy, reduced stress, improved ability to manage distractions, and improved well-being (Davis and Hayes, 2011). Mindfulness thus purifies the mind and helps people see the truths of nature or wisdom, inspires human strength and positive emotions, and at the same time helps people understand themselves and others, which enables them to improve their spiritual world and have a better understanding of others, which is the main concept of workplace spirituality (Petchsawang and McLean, 2017).

According to Gupta et al. (2014), many major corporations, including IBM and Microsoft, have used meditation, spiritual lectures, and yoga sessions to enhance their employees’ workplace spirituality. These lessons exemplify the role of mindfulness, which helps people be in the present moment and helps people connect with others and engage in work (Federman, 2009). In fact, by paying attention to and being aware of current experiences, mindful individuals are able to observe rather than identify with the contents of consciousness (i.e., thoughts, emotions, feelings, and judgments), but see them in a clearer and more objective way, able to relate the self to negative affective dissociation at work (Baer et al., 2006; Hülsheger et al., 2014), thereby keeping the mind pure. From the perspective of resource conservation theory, mindfulness can be viewed as a cognitive resource and a competency resource. Dollard and Bakker (2010) have shown that resources in organizational settings include working conditions, job rewards, social support, employment opportunities and personal energy etc. Mindfulness can improve employees’ personal energy, providing the energy resources needed at work, so that they can focus more on the current work (Montani et al., 2018). As a result, mindfulness training in organizations becomes more meaningful. Scholars mention that practicing mindfulness meditation has positive effects on psychological, spiritual, physical, and organizational benefits. Petchsawang and McLean (2017) empirically study the mechanism of the relationship between workplace spirituality and work engagement by establishing a link between mindfulness, mental outcomes (workplace spirituality) and organizational outcomes (work engagement). Liu et al. (2020b) stated that mindfulness can boost workplace spirituality for flight attendants, and Izak (2012) proposed that workplace spirituality can promote work engagement, and employees with workplace spirituality can find the meaning of their work. The more they feel that their work is meaningful, the more enthusiastic (dedication) they are in their work, and the more energy they have to overcome difficulties (vitality) at work, and thus become more engaged in their work. Therefore, mindfulness practice is seen as one of the tools to improve workplace spirituality, thereby improving work performance, based on which we draw the following hypotheses:

***Hypothesis 2***: Mindfulness has a significant positive effect on workplace spirituality.

### Mindfulness and digital competencies

The term “competency” was first introduced into the psychological literature in 1973 (McClelland, 1973), and it comes from Latin and means “fit” (Bueno and Tubbs, 2004). Competency refers to “an individual’s demonstrated knowledge, skill, or ability” (Ulrich et al., 1995) that activates actual performance and employee potential (Luna Arocas and Morley, 2015). Spencer and Spencer (1993) identified five categories of competency characteristics, including motivation, characteristics, self-concept, knowledge, and skills. First, motivation is the thought that a person keeps thinking or wants to stimulate action. Motivation drives, guides, and chooses behavior toward certain actions or goals and away from others. Recent research has found that mindfulness can improve job satisfaction, intrinsic motivation, and reduce emotional load (Parchment, 2022), and it is able to range from deep stimulation to conscious awareness of employees self-concept, traits, and motivational abilities, which are also important predictors of competency.

With the transformation of the current era, the market has developed many new industries, such as the new media industry, which are specially built on the basis of digitalization, and will even gradually form the entire online world, even the “traditional” industries have experienced a huge digital revolution (Bitay and Kovács, 2010). Therefore, in these emerging industries, traditional competencies should be transformed into digital competencies in the new era, and the new skill requirement for web editors should be digital competencies. Digital competencies is defined as “exploring and confronting new technological situations in a flexible manner, analyzing, selecting and critically evaluating data and information, exploiting the potential of technology to represent and solve problems, building shared and collaborative knowledge, and at the same time cultivate a sense of personal responsibility and respect for mutual rights and obligations.” As a result, new media companies are increasingly aware of the gap between the existing and required digital competencies of web editors (Oberländer et al., 2020), and improving the digital competencies of web editors to meet the challenges of working in a digital future has become a new media company’s current development strategy. Ngo et al. (2020) stated that mindfulness is a state of being able to actively focus on the present moment and practice acceptance without judgment. Mindfulness for web editors can help them become aware of digital transformation trends and focus on assessing their own job competencies in the moment. Digital competencies is a new skill that web editors need to grow, and mindfulness can foster greater awareness among employees of internal experiences and intuitions they may not be aware of. Web editors with this awareness are more likely to continuously learn, thereby increasing the competencies required in their work (Gawande et al., 2019), it means increasing digital competencies for web editors. Therefore, we can assume that:

***Hypothesis 3***: Mindfulness has a significant positive effect on digital competencies.

### Workplace spirituality and work performance

Over the past few decades, workplace spirituality has drawn increased attention, especially its impact on job outcomes (Petchsawang and Duchon, 2012; Petchsawang and McLean, 2017). The concept of workplace spirituality is not a new one, as it is based on organizational and management theory (Driscoll and Wiebe, 2007). Workplace spirituality is defined as the organisational encouragement of employees’ work-related spirituality, which focuses on meaning, community, and connection (Ashmos and Duchon, 2000). Employees crave a deeper sense of purpose, they are more motivated by spiritual than material benefits, and they voluntarily put more effort and time into achieving various tasks (Kolodinsky et al., 2008). Past research has shown that the myriad benefits of workplace spirituality include reduced deviance, increased job satisfaction, increased employee engagement, improved innovative behaviors, and improved work performance (Jnaneswar and Sulphey, 2021). Work performance is the efficiency with which employees complete their work and contributes to the overall success of the organization. Work performance includes two main factors, task performance and situational performance. Among them, situational performance refers to discretionary additional role behavior beyond formal job responsibilities (such as guiding colleagues), and is an important component of work performance (Huang et al., 2022). According to the concept of workplace spirituality, employees with high workplace spirituality are willing to try to help their colleagues to relieve pain, and can easily put themselves in others’ shoes (Petchsawang, 2008). These behaviors will help to improve the situational performance of employees and thus improve the overall performance of the organization.

In addition, workplace spirituality goes beyond the nature of interesting and satisfying work into the spiritual horizon of work, which involves finding deeper meaning, purpose, and being satisfied with one’s work (Petchsawang and McLean, 2017). The spillover theory states that when people are content in their spiritual lives, their satisfaction transfers to their professional lives (Kolodinsky et al., 2008), and when they are happy at work, they become more engaged in their work. And, developing mental strength in the workplace influences employees’ positive perceptions of the organization. In fact, when employees realize that their organizational support fosters their spiritual well-being, they are more likely to be engaged and put more effort into their work (Saks, 2006).

Researchers have provided empirical evidence that workplace spirituality, as a mixture of personal experience and organizational background, is positively associated with important organizational variables, such as job satisfaction (Lee et al., 2003; Pawar, 2009; Altaf and Awan, 2011; Gupta et al., 2014), job engagement (Milliman et al., 2003; Pawar, 2009), organizational commitment (Milliman et al., 2003; Pawar, 2009; Kazemipour et al., 2012; Gatling et al., 2016). From a theoretical point of view, organizational spiritual support for employees will improve employees creativity, motivation, commitment, and work engagement (Osman-Gani et al., 2013), and these factors are closely related to organizational performance. When organizations increase workplace spirituality and job engagement, they can expect the best results, including improved work performance. Furthermore, in addition to employee performance, Albuquerque et al. (2014) empirically report that workplace spirituality can positively influence performance at the organizational level. Additionally, Benefiel et al. (2014) demonstrated through a thorough research that workplace spirituality is favourably correlated with performance and productivity across cultures and nations. As a result, we can assume that:

***Hypothesis 4***: Workplace spirituality has a significant positive effect on work performance.

### Digital competencies and work performance

New technologies are having a significant impact on how we work, in addition to the enormous impact that our society’s ongoing digitalization is having on our daily lives (Zaphiris and Ioannou, 2016; Murawski and Bick, 2017; Nyikes, 2018). Employers’ preferred skills and the labour market are changing as a result of technological advancements, so the concept and research of competencies has been extended to digital competencies (Karsenti et al., 2020). Vathanophas (2007) stated that, when implementing effective human resource management, consideration should be given to introducing a competency building program for each job or task, as the competency of employees are usually related to their jobs and therefore to organizational performance. Most workplaces require at least basic digital competencies, with employees making greater use of digital information and communication technologies at work to increase productivity and facilitate the work. In new media companies, the digital competencies of web editors are closely related to their daily work, and these competency will have a significant impact on their work performance. Broadly, digital competencies are defined as “the set of skills required to use digital technologies confidently, critically and creatively to achieve goals in areas such as learning, work, leisure, integration or participation in society” (Ferrari, 2012). Digital competencies is closely related to the work tasks and professional development of web editors. As employees of new media companies, web editors need to meet the job requirements of the position and have digital competencies to better complete the work tasks of the position. They must use digital resources and enhance the application of new technologies to keep their professional skills updated to complete their tasks efficiently and improve work performance. Today, employees in most typical office workplaces spend most of their work time using digital devices, especially for web editors. Employees with digital competencies can use digital devices and technologies flexibly and freely at work, which is conducive to saving resources and completing work tasks efficiently and quickly. We can reasonably assume that:

***Hypothesis 5***: Digital competencies has a significant positive impact on work performance.

### Workplace spirituality and digital competencies

Existing research suggests that definitions of workplace spirituality can be divided into three camps: personal experience, organizational facilitation and a mixture of personal experience and organizational facilitation (Petchsawang and McLean, 2017). In personal experience, an individual’s experience of energy, pleasure, consistency, a sense of transcendence between personal values and meaningful job, is referred to as workplace spirituality (Kinjerski and Skrypnek, 2004). Therefore, employees with high workplace spirituality have strong self-concept, they have personal work motivation, and they will actively work on improving their work ability, such as knowledge and skills. Back in 2006, the European Parliament and Council identified digital competencies as one of eight key competencies for lifelong learning. In new media companies, web editors with high workplace spirituality have strong motivation to study and work, and will continue to acquire new abilities through lifelong learning to adapt to the rapidly changing work environment and growing job demands (Oberländer et al., 2020). Therefore, employees with high workplace spirituality will strive to improve their digital technology literacy to pursue the consistency of their personal values and meaningful work when they are in their own jobs, so as to study hard to have the digital competencies required for the position. From this we assume that:

***Hypothesis 6***: Workplace spirituality has a significant positive impact on digital competencies.

### The mediating role of workplace spirituality

Scholars Jnaneswar and Sulphey (2021) reviewed the previous literature and found that mindfulness is significantly related to workplace spirituality. Mindfulness-based stress reduction programs can improve employees’ workplace spirituality, thereby helping employees experience meaning and job satisfaction at work. Because mindfulness enables the brain to achieve a focused ideology (Payutto, 2002). In this state, a person is constantly aware of his thoughts and actions (Petchsawang and McLean, 2017). This can help employees to purify their minds, stimulate their work force and positive emotions, and make individuals more engaged in work (Saks, 2006). This shows the impact of mindfulness on workplace spirituality. In addition, workplace spirituality plays a key role in leadership, and its role in improving employees and organizational efficiency is widely recognized. Workplace spirituality is based on humanistic values (Jnaneswar and Sulphey, 2021), such as interconnectedness with others at work (human resources). At the same time, workplace spirituality can promote employees’ mental health (psychological resources), help employees achieve workplace happiness and harmony, and focus on work meaning and contribution to the organization. Therefore, building workplace spirituality in an organization can increase productivity and reduce turnover rate. According to the investment principle in the resource conservation theory, individuals must continuously invest in existing resources for the purpose of protecting their own resources from depletion, recovering quickly from resource depletion that has occurred, or acquiring new resources (Hobfoll, 1989). Therefore, in order to obtain stable interpersonal and psychological resources, employees will invest their existing resources by working hard and improving work performance. For example, Timms and Brough (2013) found that if employees are satisfied with the psychological resources in the workplace, they will dedicate their resources, such as work engagement, improve work performance, etc. This proves that workplace spirituality is beneficial to improve work performance. Based on the above analysis, we can assume that:

***Hypothesis 7***: Workplace spirituality plays a mediating role in mindfulness and work performance.

### The mediating role of digital competencies

According to research, the “beginner’s mindset” is one of the key attitudes of mindfulness, which refers to the mindset of being able to experience the present moment as a first experience (Kabat-Zinn, 2013). Beginner mentality of web editors can maintain a learning attitude towards digital knowledge and skills at all times, and continuously improve their digital competencies. This illustrates the impact of mindfulness on digital competencies. And, Liu et al. (2022e) have added loving-kindness meditation to the organization, and said that loving-kindness meditation can improve the effectiveness of knowledge acquisition (digitial knowledge), prevent knowledge loss, better allocate knowledge resources, and improve the core competitiveness of the organization. The current study found that employees job competencies are usually related to organizational performance and can improve work performance (Luna Arocas and Morley, 2015). Scholars Oberländer et al. (2020) redefine digital competencies in the context of the workplace by reviewing past definitions and frameworks of digital competencies. Digital competencies at work are a set of essential knowledge, skills, abilities, and other characteristics that enable workers to efficiently and successfully perform work tasks related to digital media at work. This definition shows that digital competencies have the characteristics that can help workers to better complete their work tasks and improve work performance. Therefore, we assume that:

***Hypothesis 8***: Digital competencies plays a mediating role in mindfulness and work performance.

According to the analysis and demonstration of the above eight hypotheses, the relationship between mindfulness and workplace spirituality, workplace spirituality and digital competencies, digital competencies and work performance are fully supported by the literature. Therefore, we can reasonably assume:

***Hypothesis 9***: Workplace spirituality and digital competencies play a chain mediating role between mindfulness and work performance.

In summary, this study constructs a chain mediation model of mindfulness, workplace spirituality, digital competencies and work performance. The hypothetical model is shown in Figure 1.

## Materials and methods

By reading a large amount of domestic and foreign literatures on employee mindfulness, workplace spirituality, digital competencies, and work performance, we organizes and summarizes them, and finds a scale with high reliability and validity, which is widely used and most suitable for web editors. Mindfulness variables, mainly refer to the 15-item scale revised by Brown and Ryan (2003); workplace spirituality variables, mainly refer to the 22-item scale designed by Petchsawang (2008); digital competencies variables, constructed by Castaño-Muñoz et al. (2017) 6-item scale; work performance variables, mainly refer to the 9-item scale developed by Rodwell et al. (1998). Because mindfulness, workplace spirituality, digital competencies, and work performance are all mature scales borrowed from the English context, the translation of the scales needs to be considered. The translation of the original questionnaire determines the items of the initial questionnaire. After determining the initial scale, in order to make the scale of each variable more suitable for the measurement of web editors, a small-scale expert interview was conducted with four experienced web editors, and some unclear expressions, repeated meaning or difficult to answer questions were deleted under the advice of experts. We developed a formal questionnaire that is more in line with Chinese expression habits and cultural background, and is more suitable for measuring web editors. The formal questionnaire contains a total of 31 items, including 10 items of mindfulness, 12 items of workplace spirituality, 3 items of digital competencies, and 6 items of work performance. The scale uses a five-point Likert scale ranging from 1 “strongly disagree” to 5 “strongly agree.” The complete questionnaire measurement items are shown in Table 1.

**Table 1:** Measurement Items.

Construct	References
Mindfulness	Brown and Ryan (2003)
I could be experiencing some emotion and not be conscious of it until some time later.	
I break or spill things because of carelessness, not paying attention, or thinking of something else.	
I find it difficult to stay focused on what’s happening in the present.	
It seems I am “running on automatic” without much awareness of what I’m doing.	
I rush through activities without being really attentive to them.	
I get so focused on the goal I want to achieve that I lose touch with what I am doing right now to get there.	
I do jobs or tasks automatically, without being aware of what I’m doing.	
I find myself preoccupied with the future or the past.	
I find myself doing things without paying attention.	
I snack without being aware that I’m eating.	
Workplace Spirituality	Petchsawang (2008)
Compassion	
I can easily put myself in other people’s shoes.	
I am aware of and sympathize with others.	
I try to help my coworkers relieve their suffering.	
I aware of my coworkers’ needs.	
Meaningful Work	
I experience joy in my work.	
I look forward to coming to work most days.	
My spirit is energized by my work.	
I understand what gives my work personal meaning.	
The work I do is connected to what I think is important in life.	
Transcendence	
At times, I experience an energy or vitality at work that is difficult to describe.	
At times, I experience happiness at work.	
At moments, I experience complete joy and ecstasy at work.	
digital competencies	Castaño-Muñoz et al. (2017)
I can conduct an internet search using one or more keywords.	
I can judge the reliability of a website.	
I can participate in an online chat session.	
Work Performance	Rodwell et al. (1998)
I am currently working at my best performance level.	
Employees should only do enough to get by.	
I try to be at work as often as I can.	
My work is always of high quality.	
I set very high standards for my work.	
I am proud of my work performance.	

The participants in this study are web editors working in new media companies in China. After the formal questionnaire was confirmed, we created a formal questionnaire on the Tencent questionnaire platform,^1^ and used the formal questionnaire to conduct large-scale surveys. In the process of collecting large sample data, the method of convenience sampling was adopted. First, the questionnaires were sent privately to WeChat friends who are professional web editors, and they were invited to help fill in the questionnaires. A total of 40 questionnaires were received. Then, through the Tencent questionnaire platform, questionnaires were distributed to web editors across the country, and a total of 460 questionnaires were distributed. After excluding incomplete, pattern responses and quick responses, 431 valid data were retained for the final analysis, and the response rate of the questionnaire was 86.2%.

## Data analysis

The research uses SPSS25.0 and Mplus8.3 to conduct statistical analysis on 431 valid samples to understand the relationship between the mindfulness, workplace spirituality, digital competencies and work performance variables of web editors, and to verify the research model and research hypothesis proposed in this study. Firstly, the reliability assessment is carried out by reliability analysis, construct validity test, discriminant validity test and common method bias analysis; then, structural equation model analysis is carried out to test whether the 9 groups of hypotheses in this study can be supported by empirical data; Finally, a mediation effect analysis was carried out to test whether the three groups of mediation hypotheses could be supported by empirical data.

### Demographic characteristics

In terms of obtaining basic personal information, the problems mainly relate to gender, age, marriage, education and years of work. Among the sample of participants in this study, 51.3% were male and 48.7% were female. About 44.8% of the respondents were between the ages of 26 and 35, 4.4% were over the age of 55, and 69.4% were married. In addition, 52.9% of the sample has an undergraduate education, and only 3.5% are high school or less, 43.4% have worked for more than 8 years, and 7.7% have worked for less than 1 year. The details of the respondents are shown in Table 2 below.

**Table 2:** Characteristics of respondents.

Variables	Characteristics	Frequency(n = 431)	(%)
Gender	Male	221	51.3
	Female	210	48.7
Age	18-25 years old	53	12.3
	26-35 years old	193	44.8
	36-45 years old	121	28.1
	46-55 years old	45	10.4
	55 or older	19	4.4
Marriage	unmarried	129	29.9
	Married	299	69.4
	other	3	0.7
Education levels	High school or less	15	3.5
	Technical school	90	20.9
	Undergraduate	228	52.9
	Graduate or more	98	22.7
Working years	1 year or less	33	7.7
	1-3 years	80	18.6
	4-8 years	131	30.4
	8 years or more	187	43.4

### Reliability analysis

This study uses SPSS25.0 software to conduct reliability analysis and test of each variable to ensure the reliability and consistency of the formal questionnaire. First, the overall Cronbach’s alpha value of the 31 measurement items in this study was measured, and the measured result was 0.935, which indicated that the measurement items of the overall scale in this study had good internal consistency, thus showing good reliability. Then, the reliability of the four latent variables was analyzed separately, and the Cronbach’s alpha value of mindfulness was 0.920, and the Cronbach’s alpha value of workplace spirituality was 0.912 (the Cronbach’s alpha value of the three dimensions of COM/MW/TRA was 0.912, 0.919, 0.880), the Cronbach’s alpha value of digital competencies is 0.760, and the Cronbach’s alpha value of work performance is 0.8610. This indicates that the reliability of each latent variable is good, and further analysis can be carried out.

### Construct validity analysis

In this study, the robust maximum likelihood estimation (MLR) was used in the Mplus8.3 software to test the construct validity of the six latent variable measurement scales using confirmatory factor analysis. The confirmatory factor analysis model is known as a general structural equation model (SEM) measurement model. The measurement model and the structural equation model are consistent with the evaluation index and evaluation standard of the degree of fit, mainly including: χ^2^ (chi-square), df (degree of freedom), the smaller the ratio of χ^2^ to df, the better the fit of the model. Schumacker and Lomax (2004) considered a ratio of χ^2^ to df below 5 to be acceptable. In this study, the ratio of χ^2^ to df was 1.15, which indicated that the model fit in this study was very good. Asymptotic residual mean square and square root (RMSEA), Browne (1992) believed that when the RMSEA value was lower than 0.05, the model fit was good, and when the RMSEA value was lower than 0.1, the model fit was reasonable (Wu, 2009, Structural Equation Modeling). In this study, the RMSEA value was 0.019, which was lower than 0.01, indicating that the model fit in this study was reasonable. In addition, comparing the fit index (CFI), Bentler (1990) believed that the CTI value was greater than 0.9, indicating that the model fit was good. Non-standard fit index (TLI), Bentler and Bonett (1980) considered that the TLI value was greater than 0.9, indicating that the model fit was better. The standard residual mean square and square root is SRMR, and the general standard is SRMR<0.1, the smaller the better (Wu, 2009, structural equation model). The data of this study showed that CFI = 0.992, TLI = 0.991, SRMR = 0.029, all in line with the ideal indicators.

In the analysis of the measurement model, the indicators to observe the aggregate validity mainly include the average variance extracted (AVE). In general, the larger the AVE value, the stronger the commonality of the measurement items, the more it can reflect the same variable, and the higher the aggregation validity. Fornell and Larcker (1981) believed that the convergent validity was ideal if the AVE value was greater than 0.5. In this research model, the minimum AVE value is 0.510, and the others are higher than this value, indicating that the convergent validity of the research model has reached ideal. Secondly, the composite reliability (CR) is one of the criteria for judging the intrinsic quality of each measurement item. If the composite reliability value of each measurement item is greater than 0.6, it corresponds to the CR value of the study shown in Table 3. The minimum CR value is 0.759, indicating that the internal consistency of each structure was achieved, and it also showed that the internal consistency of the measurement scale was better. These data fully indicate that the model is well fitted, and further analysis can be carried out.

**Table 3:** Results of the measurement model.

Construct	Indicators	Factor loading	S.E.	Est./S.E	*p*-value	CR	AVE
Mindfulness	MF1	0.745	0.024	31.442	***	0.921	0.537
	MF2	0.706	0.026	26.747	***		
	MF3	0.749	0.023	31.957	***		
	MF4	0.747	0.024	31.791	***		
	MF5	0.728	0.025	29.262	***		
	MF6	0.742	0.024	30.984	***		
	MF7	0.754	0.023	32.685	***		
	MF8	0.717	0.026	27.967	***		
	MF9	0.726	0.025	29.059	***		
	MF10	0.714	0.026	27.695	***		
Compassion	COM1	0.874	0.015	59.022	***	0.912	0.723
	COM2	0.857	0.016	53.756	***		
	COM3	0.844	0.017	50.269	***		
	COM4	0.825	0.018	45.094	***		
Meaningful Work	MW1	0.855	0.016	54.555	***	0.919	0.696
	MW2	0.848	0.016	52.496	***		
	MW3	0.847	0.016	52.207	***		
	MW4	0.799	0.020	40.450	***		
	MW5	0.820	0.018	44.800	***		
Transcendence	TRA1	0.872	0.017	50.829	***	0.883	0.716
	TRA2	0.887	0.016	53.816	***		
	TRA3	0.775	0.023	33.892	***		
digital competencies	DC1	0.698	0.034	20.463	***	0.760	0.514
	DC2	0.738	0.033	22.599	***		
	DC5	0.714	0.034	21.245	***		
Work Performance	WP1	0.760	0.025	30.990	***	0.862	0.510
	WP2	0.726	0.027	27.233	***		
	WP3	0.682	0.030	23.114	***		
	WP5	0.694	0.029	24.173	***		
	WP6	0.718	0.027	26.348	***		
	WP7	0.705	0.028	25.122	***		
Workplace Spirituality(second order)	COM	0.734	0.034	21.567	***	0.759	0.513
	MW	0.733	0.034	21.521	***		
	TRA	0.680	0.037	18.190	***		
χ^2^/df = 488.371/425;RMSEA = 0.019;CFI = 0.992;TLI = 0.991;SRMR =0.029.							

*** indicates that the factor loading of the measurement item is significant when the standard line *p* < 0.001.

### Discriminant validity test

Discriminant validity refers to the degree to which a latent variable is truly different from other latent variables according to the criteria of empirical research, and any measurement item should reflect only one latent variable. Use SPSS25.0 to test the correlation coefficient matrix of each variable in the sample data of the formal questionnaire, and calculate the AVE square root of each variable. The results are shown in Table 4. It can be seen from Table 4 that the correlation coefficient between the six latent variables is between 0.290 and 0.499, none of them are 1, and they all reach a significant level. In addition, most of the correlation coefficients between all variables in this study and another variable are less than the square root of the AVE value of the variable, indicating that the six variables in this study have good discriminant validity.

**Table 4:** Correlation coefficient matrix and square root of AVE value of each variable in the formal questionnaire.

Constructs	MF	COM	MW	TRA	DC	WP
MF	0.537					
COM	0.421**	0.723				
MW	0.425**	0.491**	0.696			
TRA	0.356**	0.444**	0.462**	0.716		
DC	0.309**	0.290**	0.291**	0.239**	0.514	
WP	0.492**	0.483**	0.461**	0.448**	0.499**	0.510

The shaded value on the diagonal is the square root of the AVE value.

** Indicates a significant correlation at the 0.01 level (two-tailed).

### Common method bias

Common method bias may seriously confuse research results and potentially mislead research conclusions, which is a systematic error (Xiong et al., 2012). Common method bias exists widely in researches using questionnaires and psychology, so it is necessary to test the common method bias for measurement items.

This study has adopted a variety of methods to reduce the common method bias in the research design stage of the questionnaire, such as interview method, expert opinion consultation method and so on. However, most of the measurement data of the six main variables in this study are obtained from the survey on the Tencent platform, and there may be common method bias. Therefore, this study needs to test whether the data of the questionnaire survey has the problem of common method bias.

There are many ways to test the common method bias, and the Harman single factor method is the most commonly used method by scholars to test the common method bias (Tang and Wen, 2020). In this study, Mplus 8.3 software was used to test the common method deviation of the questionnaire data by Hannan’s single factor method. The results are shown in Table 5. From Table 5, χ^2^/df = 8.632 > 5, RMSEA = 0.133 > 0.1, CFI = 0.568 < 0.9, TLI = 0.537 < 0.9, SRMR = 0.109 > 0.08. According to the test results, the fitness indicators of the Harman single-factor model are inconsistent with the standard value of the fitness of the structural equation model, and the fit of the single-factor structural model is very poor, which shows that the measurement items of each variable in this study are no risk of common method bias, which does not need to be controlled.

**Table 5:** Common method bias test (N = 431).

Model	χ^2^	df	χ^2^ /df	RMSEA	CFI	TLI	SRMR
Hannan	3746.221	434	8.632	0.133	0.568	0.537	0.109

## Measurement model

### Structural equation model

When the evaluation indexes and standards of the fitness of the above structural equation models reach the ideal value, the fitting degree of the model in this study is acceptable and suitable for structural equation model analysis. Structural Equation Modeling (SEM) is a powerful statistical technique that combines measurement models (confirmative factor analysis) and structural models (regression analysis or path analysis) together with statistical tests to test and compare theoretical models and ideal technique for refining and testing construct validity (Garver and Mentzer, 1999). This study uses structural equation model to test and analyze all hypotheses.

As stated in the theoretical framework, the purpose of using structural equation model analysis is to test whether nine sets of hypotheses can be supported by empirical data, including (1) mindfulness has a significant positive effect on work performance. (2) Mindfulness has a significant positive impact on workplace spirituality. (3) Mindfulness has a significant positive impact on digital competencies. (4) Workplace spirituality has a significant positive impact on work performance. (5) Digital competencies has a significant positive impact on work performance. (6) Workplace spirituality has a significant positive impact on digital competencies. (7) Workplace spirituality plays a mediating role between mindfulness and work performance. (8) Digital competencies plays a mediating role between mindfulness and work performance. (9) Workplace spirituality and digital competencies play a chain mediating role between mindfulness and work performance. This study intends to use Mplus8.3 to test the constructed research model.

### Structural equation model fit

Ensuring that the fitting degree between the proposed research model and the data of each measurement variable conforms to the evaluation index and standard of the structural equation model fitting degree is the premise of structural equation model analysis. This study uses Mplus8.3 to test the constructed structural equation model, and obtains that the specific model fitting index measurement model is consistent, indicating that the fitting index meets the requirements of the structural equation model, which indicates that the research on the relationship between latent variables proposed in this study has a good fit with the measured data and can be used for subsequent analysis.

### Summary of hypothesis testing results and path analysis of the model

**Figure 2:**
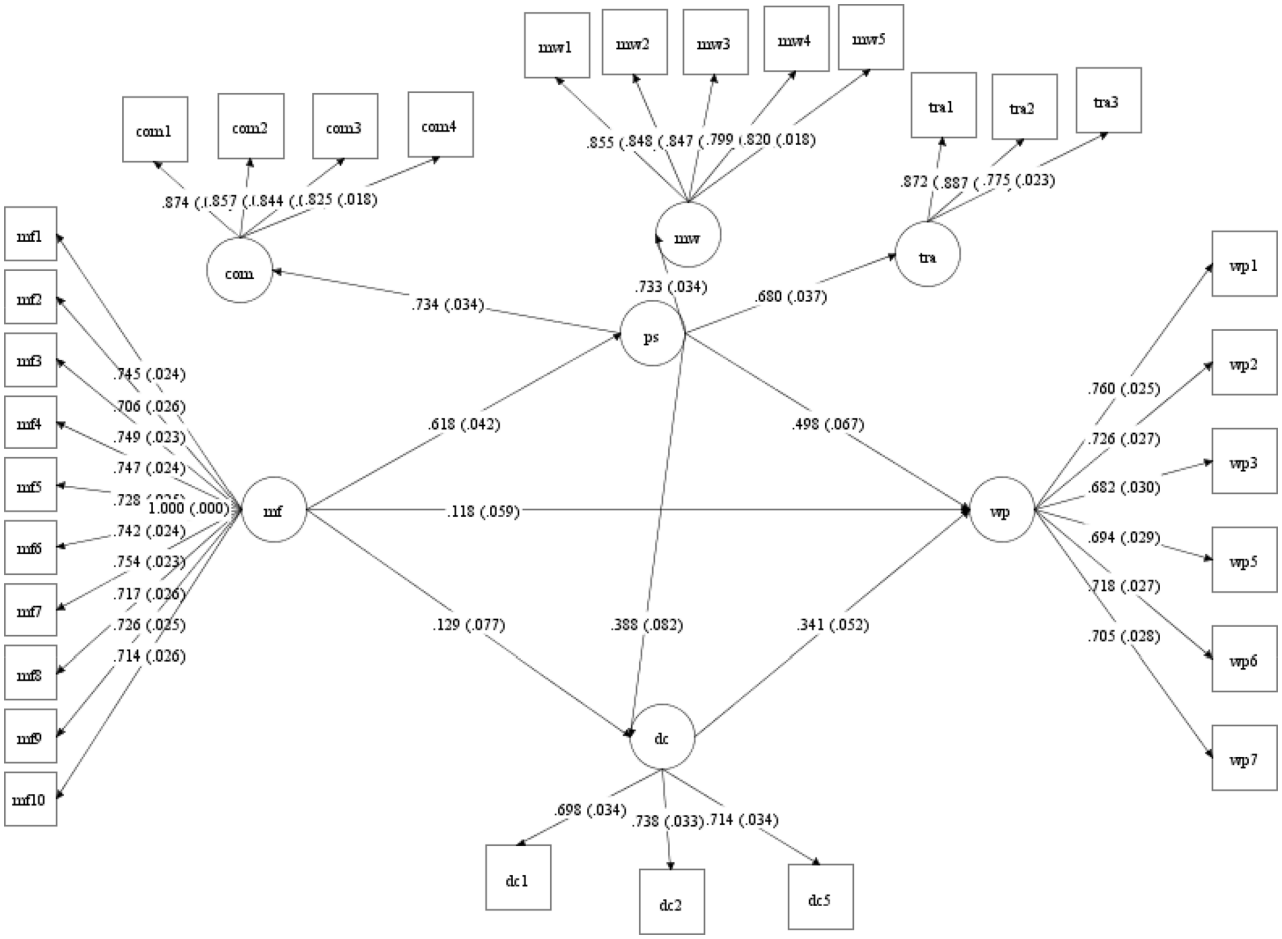
The output of the structural equation model.

The road map of the model in this study is shown in Figure 2, and the results of the hypothesis validation results are summarized in Table 6.

**Table 6:** Structural equation model analysis table.

Path	Standardization coefficients	S.E.	Est./S.E	*p*-Value	Results
Mindfulness→Work Performance	0.118	0.059	1.999	0.046*	Supported
Mindfulness→Workplace Spirituality	0.618	0.042	14.896	***	Supported
Mindfulness→digital competencies	0.129	0.077	1.675	0.094*	Supported
Workplace Spirituality→Work **Performance**	0.498	0.067	7.415	***	Supported
digital competencies→Work Performance	0.341	0.052	6.493	***	Supported
Workplace Spirituality→digital competencies	0.388	0.082	4.718	***	Supported
Model fit:.χ^2^ (df) = 488.371/425;RMSEA = 0.019;CFI = 0.992;TLI = 0.991; SRMR = 0.029.					

***, **, * indicate that p is significant at the level of 0.001, 0.01, and 0.1, respectively; in Mplus, Est/S.E. = *t* value.

Thus, the correlation between the latent variables can be analyzed. (1) Mindfulness and work performance. According to the output results of the structural equation model, the standardized path coefficient of mindfulness on work performance variables is 0.118, the t value is 1.999, and the *p* value is less than 0.05, indicating that mindfulness has a significant positive impact on work performance. This study assumes that hypothesis 1 is supported. It can be seen that the stronger the mindfulness of web editors at work, the higher their work performance. (2) Mindfulness and workplace spirituality. According to the output results of the structural equation model, it can be seen that the standardized path coefficient of mindfulness on the workplace spirituality variable is 0.618, the t value is 14.896, and the p value is less than 0.001, indicating that mindfulness has a significant positive impact on workplace spirituality. This study assumes that hypothesis 2 is supported. It can be seen from this that the stronger the mindfulness of web editors, the stronger their workplace spirituality. (3) Mindfulness and digital competencies. According to the output results of the structural equation model, the standardized path coefficient of mindfulness on digital competencies is 0.129, the t value is 1.675, and the p value is less than 0.01, indicating that mindfulness has a significant positive impact on digital competencies. This study assumes that hypothesis 3 is supported. It can be seen that the stronger the mindfulness of web editors, the stronger their digital competencies. (4) Workplace spirituality and work performance. According to the output results of the structural equation model, the standardized path coefficient of workplace spirituality to work performance is 0.498, the t value is 7.415, and the p value is less than 0.001, indicating that workplace spirituality has an significant positive impact on work performance, hypothesis 4 of this study is supported. It can be seen that the stronger the workplace spirituality of the web editors, the higher the work performance. (5) Digital competencies and work performance. According to the output results of the structural equation model, the standardized path coefficient of digital competencies variables to work performance variables is 0.341, the t value is 6.493, and the p value is less than 0.001, indicating that digital competencies has a significant impact on work performance. There is a significant positive effect on performance, and this study assumes that hypothesis 5 is supported. It can be seen that the stronger the digital competencies of the web editors, the higher the work performance. (6) Workplace spirituality and digital competencies. According to the output results of the structural equation model, the standardized path coefficient of workplace spirituality to digital competencies is 0.388, the *t* value is 4.718, and the *p* value is less than 0.001, indicating that workplace spirituality has a significant positive impact on digital competencies. This study suppose hypothesis 6 is supported. It can be seen from this that the stronger the workplace spirituality of web editors, the stronger their digital competencies.

### Mediation effect analysis

In this study, Mplus software is used, and the Bootstrap method is used, and the number of repeated samples is 5,000 to examine the mediation effect relationship between the workplace spirituality and digital competencies of web editors between their mindfulness and work performance. Bootstrap method is an important statistical method for interval estimation in nonparametric statistics. The Bootstrap method creates multiple simulated data sets by repeating sampling with replacement for a given data set, thereby generating a series of empirical distributions of the statistics to be tested. The Bootstrap method can calculate standard errors, construct confidence intervals and hypothesis tests can be performed on many types of sample statistics. The statistical results calculated by the Bootstrap method, if the estimated value of the indirect effect (*p*-value) is significant, and does not include 0 in the 95% confidence interval, it means that there is a mediating effect characteristic (Lau and Cheung, 2012). This study uses the Bootstrap method to test the mediation effect, and the results are shown in Table 7.

**Table 7:** Mediating effect test.

The mediation path	Indirect effects		Direct effects				Total effect		
	SE	95% confidence interval	*p*-Value	SE	confidence interval	*p*-Value	SE	confidence interval	*p*-Value
P1:Mindfulness → Workplace Spirituality → Work Performance	0.308 (0.062)	[0.221,0.424]	0.000	0.118 (0.065)	[0.041,0.126]	0.070	0.552 (0.050)	[0.466,0.628]	0.000
P2:Mindfulness → Digital competencies → Work Performance	0.044 (0.035)	[−0.007,0.107]	0.208						
P3:Mindfulness → Workplace Spirituality → Digital competencies → Work Performance	0.082 (0.026)	[0.041,0.126]	0.002						

In the mediating path “P1: Mindfulness→Workplace Spirituality→Work Performance,” the 95% confidence interval for the specific indirect effect of mindfulness on work performance is [0.221, 0.424], 0 is not included, so the mediating effect is significant; The direct effect of is significant, and the 95% confidence interval is [0.041, 0.126], 0 is not included, so the partial mediating effect of workplace spirituality between mindfulness and work performance is significant. The specific indirect effect size was 0.308, the direct effect size was 0.118, and the total effect size was 0.552. The ratio of indirect effect to total effect was 0.308/0.552 = 0.557, which indicated that when mindfulness had an impact on work performance, 55.7% of the variance was caused by workplace spirituality.

In the mediating path “P2: Mindfulness → Digital competencies → Work Performance,” the 95% confidence interval for the specific indirect effect of mindfulness on work performance is [−0.007, 0.0107], including 0, so the mediating effect is not significant, so hypothesis 8 invalid.

In the mediating path “P3: Mindfulness → Workplace Spirituality → Digital competencies → Work Performance,” the 95% confidence interval of the specific indirect effect of mindfulness on work performance is [0.041, 0.126], 0 is not included, so the mediating effect is significant; The direct effect of mindfulness on work performance is significant, and the 95% confidence interval is [0.041, 0.126], excluding 0. The chain mediating effect of workplace spirituality and digital competencies between mindfulness and work performance is significant. The specific indirect effect size between mindfulness and work performance was 0.082, the direct effect size was 0.118, and the total effect size was 0.552. The ratio of indirect effect to total effect is 0.082/0.552 = 0.149, which indicates that when the mindfulness of web editors has an impact on work performance, 14.9% of the variation is through workplace spirituality that affects digital competencies, which in turn affects their work performance.

## Discussion and conclusions

The primary objective of this study was to explore the relationship between mindfulness, workplace spirituality, digital competencies, and work performance. This study verified that mindfulness has a significant positive effect on work performance, which is consistent with previous research (Huang et al., 2022). However, the impact of mindfulness on work performance has a relatively complex mechanism, and its complex mechanism still needs to be continuously explored. This study confirms that workplace spirituality plays a strong mediating role in the relationship between web editors’ mindfulness and work performance. When web editors’ mindfulness has an impact on work performance, 55.7% of the variation is caused by their workplace spirituality. Workplace spirituality and digital competencies play a chain mediating role between web editors’ mindfulness and work performance. When web editors’ mindfulness has an impact on work performance, 14.9% of the variation is that digital competencies is affected by workplace spirituality, and then affect their work performance, which shows that employees with high mindfulness will be more likely to focus. They will focus more on the current work and awareness, spiritually think the current work is meaningful and valuable, and are committed to continuous improvement of their own job skills to improve work performance.

Workplace spirituality is largely based on the mindfulness of web editors. This study confirmed that mindfulness has a significant and positive impact on the workplace spirituality of web editors, with a path coefficient of as high as 0.618 (*p* < 0.001), indicating that mindfulness is an important antecedent variable of workplace spirituality. This is in line with Payutto's (2002) view that practicing mindfulness meditation trains the brain and brings it to a fully focused state of consciousness, which increases employees’ workplace spirituality, allowing employees to discover the meaning of their work. They will be passionate about their work, and dedicate themselves to work (Petchsawang and McLean, 2017), thereby improving work performance.

Finally, the study found that the impact of workplace spirituality on work performance is greater than the impact of digital competencies on work performance. The results of this study show that the workplace spirituality of web editors has a significant positive impact on work performance, with a standardized coefficient of 0.498 (*p* < 0.001); digital competencies has a significant positive impact on work performance with a standardized coefficient of 0.341 (p < 0.001), which indicates that the workplace spirituality of web editors has a greater impact on work performance than digital competencies. This confirms from the side that for a group of web text workers such as web editors, they identify with the values of groups and organizations and the meaning of work in their work, and then surpass themselves and generate a sense of interconnectedness. Experience has a great role in promoting the improvement of their work performance. As a professional skill of web editors, digital competencies also plays a very important role in today’s labor market and has a significant positive impact on work performance. However, compared with the spiritual resource of workplace spirituality possessed by web editors, its importance is relatively low.

## Theoretical contributions

Numerous studies have confirmed the validity of the JD-R theoretical model, but limited research has been done on mindfulness, workplace spirituality, digital competencies, and work performance in web editors. Therefore, we use the JD-R theoretical model to make some breakthroughs in theoretical contributions. Firstly, the concrete representation of work resources in the JD-R model is extended. From the perspective of research model construction, based on the JD-R model, this research classifies “mindfulness” as an individual’s intrinsic work resource, and believes that it can generate intrinsic motivation, prompting web editors to improve workplace spirituality, and thus produce good organizations result. The results of the empirical study confirmed the hypothesis that when web editors’ mindfulness had an impact on work performance, 55.7% of the variance was caused by their workplace spirituality. This suggests that it is appropriate to consider mindfulness as an individual work resource, extending the specific representation of work resources in the JD-R model. Based on the motivation-driven process of the JD-R model, this research constructs a chain mediation model of “mindfulness-workplace spirituality-digital competencies-work performance” for web editors, which is supported by empirical research. This shows that mindfulness, as an internal resource of an individual, has a very complicated mechanism to improve work performance by motivating professional individuals. Professional individuals with high mindfulness tend to identify with the meaning and value of work at the spiritual level, have more good inner experience of work, and are committed to continuously improving their work skills, thereby improving work performance. At the same time, it also shows that it is appropriate and effective to use the JD-R model to study the multiple and interactive influencing factors of Chinese web editors’ work performance, which verifies and expands the applicable boundaries and application scenarios of the JD-R theoretical model.

Second, it confirms that the mechanism of mindfulness’s impact on work performance is quite complex. Previous studies have shown positive effects of mindfulness on work performance and other outcomes in the workplace, suggesting that mindfulness can positively impact workplace performance, relationships, well-being, and job satisfaction (Glomb et al., 2011; Dane and Brummel, 2014; Hyland et al., 2015). This study is consistent with previous theoretical and empirical evidence that mindfulness has a positive effect on improving work performance (Good et al., 2016). Ngo et al. (2020) reviewed the past literature and indicated that few scholars have simultaneously studied the indirect effects of mindfulness on work performance. Our study goes a step further by exploring the entire impact mechanism of mindfulness, examining how workplace spirituality and digital competencies, separately and together, indirectly affect work performance. This somewhat expands the existing literature in the field of mindfulness. In addition, we also provide a new research perspective. Different from the previous research on mindfulness of factory employees and mindfulness of medical clinic employees, the subject of our research is web editors, and almost no research has been carried out on this subject. The more challenging and distracting nature of the work of web editors makes mindfulness even more important in this working group.

In addition, compared to other groups, the mindfulness of web editors has a greater impact on workplace spirituality. After empirical testing, this study found that the path coefficient of the influence of mindfulness on web editors’ workplace spirituality reached 0.618 (p < 0.001), that is, the higher the mindfulness of web editors, the higher their workplace spirituality, and there is a strong relationship between the two variables. This is in line with the existing literature (Petchsawang and McLean, 2017) studying the population of education, public health services and industrial organizations in Bangkok, which has demonstrated a positive relationship between mindfulness and workplace spirituality. The path coefficient r is 0.39 (*p* = 0.01) have some differences. This suggests that the mindfulness of writers such as web editors has a greater impact on their workplace spirituality than people in education, public health services, and industrial organizations. Web editors with high mindfulness will be more awake, focused, concentrate on thinking about problems, and be aware of their emotions and consciousness, which will greatly promote them to find deeper work meaning, purpose, value, etc., and produce better work experience.

Finally, there are few current research literatures on digital competencies, most of which focus on the teaching of digital competencies in colleges and universities, and are rarely used in organizations. Today, digital competencies is closely related to the professional development of employees (Karsenti et al., 2020), and research in organizations is urgent. Through literature collection, we found that there is a lack of existing valid scales for digital competencies, and our research can further validate the validity of digital competencies scales in professional settings (Castaño-Muñoz et al., 2017), which will expand research on digital competencies boundaries, thereby promoting the widespread use of digital competencies scales. In addition, digital competencies does not have a mediating effect between mindfulness and work performance, which also makes a major breakthrough.

## Practical implications

Taken together, these results demonstrate the impact of employees mindfulness on workplace spirituality, digital competencies, and work performance. As such, mindfulness has been recommended in organizations as a potential remedy (FitzGerald, 2020) to help employees focus on their work in the moment through mindfulness practices in the organization. Organizations should bundle mindfulness practices into company training programs (Hülsheger et al., 2013; Kabat-Zinn, 2013), such as the use of lectures for thought guidance, discussions to generate thought exchanges, and meditation exercises to reinforce mindfulness (Chen et al., 2022b) and so on, help employees grow, improve work focus, and ultimately the focus of employees can be transformed into improved employees follow-up work performance. Liu et al. (2022a) investigated animated guided mindfulness meditation can increase flow ergonomics for flight attendants. New media companies can learn from this practice and use animation to guide the mindfulness of web editors. At the same time, individual web editors should also have the awareness of mindfulness exercises, and turn these exercises into their own personal resources to stimulate the positive impact of the workspace (Huang et al., 2022), reduce their negative work emotions, and improve work happiness. Considering the current COVID-19 pandemic, web editors are working from home more often, so new media companies should also start exploring ways to improve employee mindfulness through online training.

In addition, the positive influence of workplace spirituality needs to be paid attention to. In today’s technologically complex and highly competitive contemporary business environment, new media companies need to fully consider the spiritual needs of web editors, combine the company’s own characteristics, and learn from the workplace spirituality programs implemented by organizations such as Apple, HP, and Ford to improve employees’ awareness and skills (Jnaneswar and Sulphey, 2021). Organizations should pay attention to how to create a humanistic environment, treat employees at a spiritual level (Cacioppe, 2000), pay attention to employees’ needs at a deep level, support employees’ continuous learning and growth, and meet employees’ psychological needs in the workplace. Specifically, new media companies should consider meditation classes and follow-up programs to motivate employees to practice meditation as a routine to purify their minds (Petchsawang and McLean, 2017). For example, new media companies can increase their workplace spirituality by allowing employees to practice meditation in the office for 10–15 min before starting work and at the end of the workday to help them maintain meaning and enthusiasm for their work. In addition to the spiritual plan, meaningful work should be designed according to the organizational environment, promote the alignment of organizational goals with personal goals, and promote employees learning, which is also an important factor in workplace spirituality.

Finally, by examining the relationship between mindfulness and digital competencies, and the relationship between digital competencies and work performance, we also recommend that companies focus on digital competencies. Digital competencies is crucial in today’s world (Karsenti et al., 2020), it is closely tied to fast-paced technological advancements and evolves with the implementation of new tools, technologies and applications that change the way we work. Therefore, in the face of the ever-changing market environment, new media companies should carry out flexible, dynamic and professional vocational skills training and learning to help web editors learn to use digital resources and technology to solve work problems to meet the needs of future work. In addition to this, HR managers can add specific digital competencies based on web editors’ job profiles, identifying the job requirements and development potential of current and future employees.

## Limitations and future research

Although the findings of this study provide a very important contribution, there are still some limitations worth discussing. First, our research was conducted in new media companies, and we do not rule out the possibility that specific corporate environments and cultures influence exercises between mindfulness, workplace spirituality, digital competencies, and work performance. Therefore, future research should attempt to replicate and extend existing findings across different enterprises.

Second, our study extends the study of mindfulness and work performance from a new perspective, adding two variables, workplace spirituality and digital competencies, but digital competencies play a lesser role in structure. In the future, it can continue to expand its research boundaries and explore the mediating mechanism of other variables, such as work engagement, creativity, etc.

In addition, there are some limitations regarding the collection of data. Questionnaires were distributed through the Tencent questionnaire platform in this study, and there may be data quality problems. Future research can collect data from multiple sources, such as contacting specific new media companies, and issuing questionnaires to employees through internal emails from HR managers to improve the accuracy of the questionnaires.

Finally, the focus of this study is primarily at the individual level, focusing on individual employees mindfulness, workplace spirituality, digital competencies, and individual work performance, without linking them to overall organizational performance. Therefore, future research can view mindfulness as a collective phenomenon, termed mindful organization (Sutcliffe et al., 2016), or link organizational effects to existing individual-level work performance, both for the organization as a whole and for future research may be more useful.

## Data availability statement

The original contributions presented in the study are included in the article/supplementary material, further inquiries can be directed to the corresponding author/s.

## Ethics statement

Ethical review and approval was not required for the study on human participants in accordance with the local legislation and institutional requirements. Written informed consent from the patients/ participants or patients/participants legal guardian/next of kin was not required to participate in this study in accordance with the national legislation and the institutional requirements.

## Author contributions

J-ZH: Put forward the research ideas, design the research plans, analyze the data of the questionnaires, participate in the writing of the manuscript, and participate in the revision of the manuscript. X-WL: Write the manuscript, revise the manuscript according to expert opinions, and participate in the design of research ideas and research plans. H-YW: Participate in the design of research ideas and research plans, and participate in the data analysis of questionnaires. Z-WX: Participate in the design of research ideas and research plans, and participate in the revision of the manuscript. All authors contributed to subsequent revisions and approved the final manuscript.

## Funding

Achievement of a General Program supported by the National Social Science Fund of China in 2021 (No. 21BXW085) and Academic research achievement of project No. I00520-2112-592 funded by Macau Foundation.

## Conflict of interest

The authors declare that the research was conducted in the absence of any commercial or financial relationships that could be construed as a potential conflict of interest.

## Publisher’s note

All claims expressed in this article are solely those of the authors and do not necessarily represent those of their affiliated organizations, or those of the publisher, the editors and the reviewers. Any product that may be evaluated in this article, or claim that may be made by its manufacturer, is not guaranteed or endorsed by the publisher.

## Footnotes

^1^ http://wj.qq.com

## References

[ref1] Albuquerque I. F. Cunha R. C. Martins L. D. Sá A. B. (2014). Primary health care services: workplace spirituality and organizational performance. J. Organ. Chang. Manag. 27, 59–82. doi: 10.1108/JOCM-11-2012-0186, PMID: 27409075

[ref2] Altaf A. Awan M. A. (2011). Moderating affect of workplace spirituality on the relationship of job overload and job satisfaction. J. Bus. Ethics 104, 93–99. doi: 10.1007/s10551-011-0891-0

[ref3] Ashmos D. P. Duchon D. (2000). Spirituality at work: a conceptualization and measure. J. Manag. Inq. 9, 134–145. doi: 10.1177/105649260092008

[ref5] Baer R. A. Smith G. T. Hopkins J. Krietemeyer J. Toney L. (2006). Using self-report assessment methods to explore facets of mindfulness. Assessment 13, 27–45. doi: 10.1177/1073191105283504, PMID: 16443717

[ref6] Bakker A. B. Demerouti E. (2007). The job demands-resources model: state of the art. J. Manag. Psychol. 22, 309–328. doi: 10.1108/02683940710733115, PMID: 31861812

[ref7] Baytos K. Kleiner B. H. (1995). New developments in job design. Bus. Credit 97:22.

[ref8] Benefiel M. Fry L. W. Geigle D. (2014). Spirituality and religion in the workplace: history, theory, and research. Psychol. Relig. Spiritual. 6:175. doi: 10.1037/a0036597

[ref9] Bentler P. M. (1990). Comparative fit indexes in structural models. Psychol. Bull. 107:238. doi: 10.1037/0033-2909.107.2.238, PMID: 2320703

[ref10] Bentler P. M. Bonett D. G. (1980). Significance tests and goodness of fit in the analysis of covariance structures. Psychol. Bull. 88:588. doi: 10.1037/0033-2909.88.3.588, PMID: 30383824

[ref11] Bishop S. R. Lau M. Shapiro S. Carlson L. Anderson N. D. Carmody J.. (2004). Mindfulness: a proposed operational definition. Clin. Psychol. Sci. Pract. 11, 230–241. doi: 10.1093/clipsy.bph077

[ref12] Bitay E. Kovács T. (2010). “The effect of the laser surface treatments on the wear resistance” in Materials science forum. *Vol. 649*. eds. Roósz A. Mertinger V. Barkóczy P. Hoó C. (Trans Tech Publications Ltd.), 107–112.

[ref13] Brown K. W. Ryan R. M. (2003). The benefits of being present: mindfulness and its role in psychological well-being. J. Pers. Soc. Psychol. 84:822. doi: 10.1037/0022-3514.84.4.822, PMID: 12703651

[ref14] Browne M. W. (1992). Circumplex models for correlation matrices. Psychometrika 57, 469–497. doi: 10.1007/BF02294416, PMID: 11716834

[ref15] Bueno C. M. Tubbs S. L. (2004). Identifying global leadership competencies: an exploratory study. Journal of American Academy of Business 5, 80–87.

[ref16] Cacioppe R. (2000). Creating spirit at work: re-visioning organization development and leadership–part I. Leadersh. Org. Dev. J. 21, 48–54. doi: 10.1108/01437730010310730

[ref17] Castaño-Muñoz J. Kreijns K. Kalz M. Punie Y. (2017). Does digital competence and occupational setting influence MOOC participation? Evidence from a cross-course survey. J. Comput. High. Educ. 29, 28–46. doi: 10.1007/s12528-016-9123-z

[ref18] Chen H. Liu C. Zhou F. Cao X. Y. Wu K. Chen Y. L.. (2022b). Focused-attention meditation improves flow, communication skills, and safety attitudes of surgeons. Int. J. Environ. Res. Public Health 19:5292. doi: 10.3390/ijerph19095292, PMID: 35564687 PMC9099589

[ref19] Chen H. Liu C. Zhou F. Chiang C. H. Chen Y. L. Wu K.. (2022a). The effect of animation-guided mindfulness meditation on the promotion of creativity, flow and affect. Front. Psychol. 13:894337. doi: 10.3389/fpsyg.2022.894337, PMID: 35719584 PMC9204527

[ref20] Dane E. Brummel B. J. (2014). Examining workplace mindfulness and its relations to job performance and turnover intention. Hum. Relat. 67, 105–128. doi: 10.1177/0018726713487753

[ref21] Davis D. M. Hayes J. A. (2011). What are the benefits of mindfulness? A practice review of psychotherapy-related research. Psychotherapy 48:198. doi: 10.1037/a0022062, PMID: 21639664

[ref22] Deci E. L. Ryan R. M. (1985). The general causality orientations scale: self-determination in personality. J. Res. Pers. 19, 109–134. doi: 10.1016/0092-6566(85)90023-6, PMID: 32885683

[ref23] Demerouti E. Bakker A. B. Nachreiner F. Schaufeli W. B. (2001). The job demands-resources model of burnout. J. Appl. Psychol. 86, 499–512. doi: 10.1037/0021-9010.86.3.499, PMID: 11419809

[ref24] Dhiman S. Marques J. (2011). The role and need of offering workshops and courses on workplace spirituality. J. Manag. Dev. 30, 816–835. 30, 816–835. doi: 10.1108/02621711111164312

[ref25] Dollard M. F. Bakker A. B. (2010). Psychosocial safety climate as a precursor to conducive work environments, psychological health problems, and employeess engagement. J. Occup. Organ. Psychol. 83, 579–599. doi: 10.1348/096317909X470690

[ref26] Driscoll C. Wiebe E. (2007). Technical spirituality at work: Jacques Ellul on workplace spirituality. J. Manag. Inq. 16, 333–348. doi: 10.1177/1056492607305899

[ref27] Federman B. (2009). Employee engagement: A roadmap for creating profits, optimizing performance, and increasing loyalty. John Wiley & Sons. p. 256.

[ref28] Ferrari A. (2012). Digital competence in practice: An analysis of frameworks. Sevilla: JRC IPTS, 10, 82116.

[ref29] FitzGerald G. A. (2020). Misguided drug advice for COVID-19. Science 367, 1434–1434. doi: 10.1126/science.abb8034, PMID: 32198292

[ref30] Fornell C. Larcker D. F. (1981). Structural equation models with unobservable variables and measurement error: algebra and statistics. Journal of Marketing Research (JMR) 18, 39–50. doi: 10.2307/3151312

[ref31] Garver M. S. Mentzer J. T. (1999). Logistics research methods: employing structural equation modeling to test for construct validity. J. Bus. Logist. 20:33.

[ref32] Gatling A. Kim J. S. Milliman J. (2016). The relationship between workplace spirituality and hospitality supervisors’ work attitudes: a self-determination theory perspective. Int. J. Contemp. Hosp. Manag. 28, 471–489. doi: 10.1108/IJCHM-08-2014-0404

[ref33] Gawande R. To M. N. Pine E. Griswold T. Creedon T. B. Brunel A.. (2019). Mindfulness training enhances self-regulation and facilitates health behavior change for primary care patients: a randomized controlled trial. J. Gen. Intern. Med. 34, 293–302. doi: 10.1007/s11606-018-4739-5, PMID: 30511291 PMC6374253

[ref34] Glomb T. M. Duffy M. K. Bono J. E. Yang T. (2011). “Mindfulness at work” in Research in personnel and human resources management. *Vol. 30.* eds. Joshi A. Liao H. Martocchio J. J. (Bingley: Emerald Group Publishing Limited). 115–157.

[ref35] Good D. J. Lyddy C. J. Glomb T. M. Bono J. E. Brown K. W. Duffy M. K.. (2016). Contemplating mindfulness at work: an integrative review. J. Manag. 42, 114–142. doi: 10.1177/0149206315617003

[ref36] Gunaratana H. (2001). Eight mindful steps to happiness: Walking the Buddha's path. Simon and Schuster. p. 268.

[ref37] Gupta M. Kumar V. Singh M. (2014). Creating satisfied employeesss through workplace spirituality: a study of the private insurance sector in Punjab (India). J. Bus. Ethics 122, 79–88. doi: 10.1007/s10551-013-1756-5

[ref38] Hasenkamp W. Wilson-Mendenhall C. D. Duncan E. Barsalou L. W. (2012). Mind wandering and attention during focused meditation: a fine-grained temporal analysis of fluctuating cognitive states. NeuroImage 59, 750–760. doi: 10.1016/j.neuroimage.2011.07.008, PMID: 21782031

[ref39] Heaton D. P. Schmidt-Wilk J. Travis F. (2004). Constructs, methods, and measures for researching spirituality in organizations. J. Organ. Chang. Manag. 17, 62–82. doi: 10.1108/09534810410511305, PMID: 36103938

[ref40] Hobfoll S. E. (1989). Conservation of resources: a new attempt at conceptualizing stress. Am. Psychol. 44:513. doi: 10.1037/0003-066X.44.3.513, PMID: 2648906

[ref41] Huang C. C. Tu B. Zhang H. Huang J. (2022). Mindfulness practice and job performance in social workers: mediation effect of work engagement. Int. J. Environ. Res. Public Health 19:10739. doi: 10.3390/ijerph191710739, PMID: 36078454 PMC9518503

[ref42] Hülsheger U. R. Alberts H. J. Feinholdt A. Lang J. W. (2013). Benefits of mindfulness at work: the role of mindfulness in emotion regulation, emotional exhaustion, and job satisfaction. J. Appl. Psychol. 98:310. doi: 10.1037/a0031313, PMID: 23276118

[ref43] Hülsheger U. R. Lang J. W. Depenbrock F. Fehrmann C. Zijlstra F. R. Alberts H. J. (2014). The power of presence: the role of mindfulness at work for daily levels and change trajectories of psychological detachment and sleep quality. J. Appl. Psychol. 99:1113. doi: 10.1037/a0037702, PMID: 25198098

[ref44] Hyland P. K. Lee R. A. Mills M. J. (2015). Mindfulness at work: a new approach to improving individual and organizational performance. Ind. Organ. Psychol. 8, 576–602. doi: 10.1017/iop.2015.41

[ref45] Izak M. (2012). Spiritual episteme: Sensemaking in the framework of organizational spirituality. J. Organ. Chang. Manag. 25, 24–47. doi: 10.1108/09534811211199583

[ref46] Jett Q. R. George J. M. (2003). Work interrupted: a closer look at the role of interruptions in organizational life. Acad. Manag. Rev. 28, 494–507. doi: 10.2307/30040736

[ref47] Jha A. P. Morrison A. B. Dainer-Best J. Parker S. Rostrup N. Stanley E. A. (2015). Minds “at attention”: mindfulness training curbs attentional lapses in military cohorts. PLoS One 10:e0116889. doi: 10.1371/journal.pone.0116889, PMID: 25671579 PMC4324839

[ref48] Jnaneswar K. Sulphey M. M. (2021). Workplace spirituality, self-compassion and mindfulness as antecedents of employee mental wellbeing. South Asian J. Bus. Stud. doi: 10.1108/SAJBS-07-2020-0258

[ref49] Kabat-Zinn J. (2013). “Some reflections on the origins of MBSR, skillful means, and the trouble with maps” in Mindfulness (Routledge), 281–306.

[ref50] Kahn W. A. (2010). The essence of engagement: Lessons from the field. In Handbook of employeess engagement. Cheltenham, UK: Edward Elgar Publishing. doi: 10.4337/9781849806374

[ref51] Karasek R. A. Jr. (1979). Job demands, job decision latitude, and mental strain: implications for job redesign. Adm. Sci. Q. 24, 285–308. doi: 10.2307/2392498, PMID: 24274148

[ref52] Karsenti T. Poellhuber B. Parent S. Michelot F. (2020). What is the digital competencies framework?.Revue internationale des technologies en pédagogie universitaire/international journal of technologies. High. Educ. 17, 11–14. doi: 10.18162/ritpu-2020-v17n1-04

[ref53] Kazemipour F. Mohamad Amin S. Pourseidi B. (2012). Relationship between workplace spirituality and organizational citizenship behavior among nurses through mediation of affective organizational commitment. J. Nurs. Scholarsh. 44, 302–310. doi: 10.1111/j.1547-5069.2012.01456.x, PMID: 22804973

[ref54] Kinjerski V. M. Skrypnek B. J. (2004). Defining spirit at work: finding common ground. J. Organ. Chang. Manag. 17, 26–42. doi: 10.1108/09534810410511288

[ref55] Kirk U. Downar J. Montague P. R. (2011). Interoception drives increased rational decision-making in meditators playing the ultimatum game. Front. Neurosci. 5:49. doi: 10.3389/fnins.2011.00049, PMID: 21559066 PMC3082218

[ref56] Kolodinsky R. W. Giacalone R. A. Jurkiewicz C. L. (2008). Workplace values and outcomes: exploring personal, organizational, and interactive workplace spirituality. J. Bus. Ethics 81, 465–480. doi: 10.1007/s10551-007-9507-0, PMID: 27409075

[ref57] Lau R. S. Cheung G. W. (2012). Estimating and comparing specific mediation effects in complex latent variable models. Organ. Res. Methods 15, 3–16. doi: 10.1177/1094428110391673

[ref58] Lee D. J. Sirgy M. J. Efraty D. Siegel P. (2003). “A study of quality of work life, spiritual well-being, and life satisfaction” in Handbook of workplace spirituality and organizational performance, 209–230.

[ref59] Lesener T. Gusy B. Wolter C. (2019). The job demands-resources model: a meta-analytic review of longitudinal studies. Work Stress. 33, 76–103. doi: 10.1080/02678373.2018.1529065

[ref60] Liu C. Chen H. Cao X. Sun Y. Liu C. Y. Wu K.. (2022a). Effects of mindfulness meditation on doctors’ mindfulness, patient safety culture, patient safety competency and adverse event. Int. J. Environ. Res. Public Health 19:3282. doi: 10.3390/ijerph19063282 35328968 PMC8954148

[ref61] Liu C. Chen H. Liang Y. C. Hsu S. E. Huang D. H. Liu C. Y.. (2022e). The effect of loving-kindness meditation on employees’ mindfulness, affect, altruism and knowledge hiding. BMC Psychol. 10, 1–15. doi: 10.1186/s40359-022-00846-0 35644623 PMC9150317

[ref62] Liu C. Chen H. Liu C. Y. Lin R. T. Chiou W. K. (2020a). Cooperative and individual mandala drawing have different effects on mindfulness, spirituality, and subjective well-being. Front. Psychol. 11:564430. doi: 10.3389/fpsyg.2020.564430 33162908 PMC7581735

[ref63] Liu C. Chen H. Liu C. Y. Lin R. T. Chiou W. K. (2020b). Effects of loving-kindness meditation on mindfulness, spirituality and subjective well-being of flight attendants. Springer, Cham. doi: 10.1007/978-3-030-49913-6_13 PMC7349275 32560125

[ref64] Liu C. Chen H. Zhou F. Chiang C. H. Chen Y. L. Wu K.. (2022b). Effects of animated pedagogical agent-guided loving-kindness meditation on flight attendants’ spirituality, mindfulness, subjective wellbeing, and social presence. Front. Psychol. 13:894220. doi: 10.3389/fpsyg.2022.894220 35992420 PMC9382197

[ref65] Liu C. Chen H. Zhou F. Long Q. Wu K. Lo L. M.. (2022c). Positive intervention effect of mobile health application based on mindfulness and social support theory on postpartum depression symptoms of puerperae. BMC Womens Health 22, 1–14. doi: 10.1186/s12905-022-01996-4 36217135 PMC9549653

[ref66] Liu C. Chiou W. K. Chen H. Hsu S. (2022d). “Effects of animation-guided mindfulness meditation on flight attendants’ flow ergonomics” in International conference on human-computer interaction (Cham: Springer), 58–67.

[ref67] Luna Arocas R. Morley M. J. (2015). Talent management, talent mindset competency and job performance: the mediating role of job satisfaction. Eur. J. Int. Manag. 9, 28–51. doi: 10.1504/EJIM.2015.066670

[ref68] McClelland D. C. (1973). Testing for competence rather than for intelligence. Am. Psychol. 28:1. doi: 10.1037/h0034092, PMID: 4684069

[ref69] Milliman J. Czaplewski A. J. Ferguson J. (2003). Workplace spirituality and employeess work attitudes: an exploratory empirical assessment. J. Organ. Chang. Manag. 16, 426–447. doi: 10.1108/09534810310484172, PMID: 27409075

[ref70] Montani F. Dagenais-Desmarais V. Giorgi G. Grégoire S. (2018). A conservation of resources perspective on negative affect and innovative work behaviour: the role of affect activation and mindfulness. J. Bus. Psychol. 33, 123–139. doi: 10.1007/s10869-016-9480-7

[ref71] Mu G. J. (2010). On the cultivation of compound internet editing talents. Chin. Edit. 4, 84–86.

[ref72] Murawski M. Bick M. (2017). Digital competences of the workforce–a research topic? Bus. Process. Manag. J. 23, 721–734. doi: 10.1108/BPMJ-06-2016-0126

[ref73] Ngo L. V. Nguyen N. P. Lee J. Andonopoulos V. (2020). Mindfulness and job performance: does creativity matter? Australas. Mark. J. AMJ 28, 117–123. doi: 10.1016/j.ausmj.2019.12.003

[ref74] Nyikes Z. (2018). Contemporary digital competencies review. INDECS 16, 124–131. doi: 10.7906/indecs.16.1.9, PMID: 36356324

[ref75] Oberländer M. Beinicke A. Bipp T. (2020). Digital competencies: a review of the literature and applications in the workplace. Comput. Educ. 146:103752. doi: 10.1016/j.compedu.2019.103752

[ref76] Osman-Gani A. M. Hashim J. Ismail Y. (2013). Establishing linkages between religiosity and spirituality on employee performance. Empl. Relat. 35, 360–376. doi: 10.1108/ER-04-2012-0030

[ref77] Parchment J. (2022). Mindfulness: a necessary leadership competency. Nurs. Made Incred. Easy 20, 17–20. doi: 10.1097/01.NME.0000816540.82548.88, PMID: 35877301

[ref78] Pawar B. S. (2008). Two approaches to workplace spirituality facilitation: a comparison and implications. Leadersh. Org. Dev. J. 29, 544–567. doi: 10.1108/01437730810894195

[ref79] Pawar B. S. (2009). Individual spirituality, workplace spirituality and work attitudes: an empirical test of direct and interaction effects. Leadersh. Org. Dev. J. 30, 759–777. doi: 10.1108/01437730911003911

[ref80] Payutto P. A. (2002). Buddhist perspectives on economic concepts. Mindfulness in the marketplace: Compassionate responses to consumerism, 77–92.

[ref81] Petchsawang P. (2008). Workplace spirituality and Buddhist meditation. University of Tennessee. Available at: https://trace.tennessee.edu/utk_graddiss/587

[ref82] Petchsawang P. Duchon D. (2009). Measuring workplace spirituality in an Asian context. Hum. Resour. Dev. Int. 12, 459–468. doi: 10.1080/13678860903135912

[ref83] Petchsawang P. Duchon D. (2012). Workplace spirituality, meditation, and work performance. J. Manag. Spiritual. Relig. 9, 189–208. doi: 10.1080/14766086.2012.688623

[ref84] Petchsawang P. McLean G. N. (2017). Workplace spirituality, mindfulness meditation, and work engagement. J. Manag. Spiritual. Relig. 14, 216–244. doi: 10.1080/14766086.2017.1291360

[ref85] Quaglia J. T. Brown K. W. Lindsay E. K. Creswell J. D. Goodman R. J. (2015). “From conceptualization to operationalization of mindfulness” in Handbook of mindfulness: Theory, research, and practice. eds. Brown K. W. Creswell J. D. Ryan R. M. (The Guilford Press). 151–170.

[ref4] Reb J. Atkins P. (2015). Mindfulness in organizations: Foundations, research, and applications. Cambridge: Cambridge University Press. 1–148. doi: 10.1017/CBO9781107587793

[ref86] Rerup C. Levinthal D. A. (2014). “Situating the concept of organizational mindfulness: the multiple dimensions of organizational learning” in Mindful change in times of permanent reorganization (Berlin, Heidelberg: Springer), 33–48.

[ref87] Rodwell J. J. Kienzle R. Shadur M. A. (1998). The relationship among work-related perceptions, employeess attitudes, and employeess performance: the integral role of communications. Hum. Resour. Manag. 37, 277–293. doi: 10.1002/(SICI)1099-050X(199823/24)37:3/4<277::AID-HRM9>3.0.CO;2-E, PMID: 25311265

[ref88] Saks A. M. (2006). Antecedents and consequences of employeess engagement. J. Manag. Psychol. 21, 600–619. doi: 10.1108/02683940610690169, PMID: 36403137

[ref89] Schaufeli W. B. (2017). Applying the job demands-resources model : a ‘how to’ guide to measuring and tackling work engagement and burnout. Organ. Dyn. 2, 120–132. doi: 10.1016/j.orgdyn.2017.04.008

[ref90] Schaufeli W. B. Bakker A. B. (2004). Job demands, job resources, and their relationship with burnout and engagement: a multi-sample study. J. Organ. Behav. 25, 293–315. doi: 10.1002/job.248

[ref91] Schumacker R. E. Lomax R. G. (2004). A beginner's guide to structural equation modeling. New York, NY: Psychology Press.

[ref92] Shao R. Skarlicki D. P. (2009). The role of mindfulness in predicting individual performance. Can. J. Behav. Sci. 41:195. doi: 10.1037/a0015166, PMID: 36215803

[ref93] Shooshtarian Z. Ameli F. Amini Lari M. (2013). The effect of labor's emotional intelligence on their job satisfaction, job performance and commitment. Iran. J. Manag. Stud. 6, 27–43. doi: 10.22059/IJMS.2013.30123

[ref94] Siegrist J. (1996). Adverse health effects of high-effort/low-reward conditions. J. Occup. Health Psychol. 1, 27–41. doi: 10.1037/1076-8998.1.1.27, PMID: 9547031

[ref95] Siegrist J. (2002). “Effort-reward imbalance at work and health” in Historical and Current Perspectives on Stress and Health (Research in Occupational Stress and Well Being). *Vol. 2*. eds. Perrewe P. L. Ganster D. C. (Bingley: Emerald Group Publishing Limited). 261–291. doi: 10.1016/S1479-3555(02)02007-3

[ref96] Smallwood J. Schooler J. W. (2015). The science of mind wandering: empirically navigating the stream of consciousness. Annu. Rev. Psychol. 66, 487–518. doi: 10.1146/annurev-psych-010814-015331, PMID: 25293689

[ref97] Spencer L.M. Spencer S.M. (1993). Competence at work: Models for superior performance. New York: John Wiley & Sons.

[ref98] Sutcliffe K. M. Vogus T. J. Dane E. (2016). Mindfulness in organizations: a cross-level review. Annu. Rev. Organ. Psych. Organ. Behav. 3, 55–81. doi: 10.1146/annurev-orgpsych-041015-062531

[ref99] Tan C. Goleman D. Kabat-Zinn J. (2012). Search inside yourself: The unexpected path to achieving success, happiness (and world peace). New York, NY: Harper Collins.

[ref100] Tang D. D. Wen Z. L. (2020). Statistical approaches for testing common method bias: problems and suggestions. J. Psychol. Sci. 43, 215–223. doi: 10.16719/j.cnki.1671-6981.20200130

[ref101] Timms C. Brough P. (2013). I like being a teacher: career satisfaction, the work environment and work engagement. J. Educ. Adm. 51, 768–789. doi: 10.1108/JEA-06-2012-0072

[ref102] Ulrich D. Brockbank W. Yeung A. K. Lake D. G. (1995). Human resource competencies: An empirical assessment. Hum. Resour. Manag. 34, 473–495. doi: 10.1002/hrm.3930340402, PMID: 36414306

[ref103] Vathanophas V. (2007). Competency requirements for effective job performance in Thai public sector. Contemp. Manag. Res. 3:45. doi: 10.7903/cmr.49

[ref104] Wang R. (2022). A brief discussion on the professional ethics and professional accomplishments of web editors in the Omni-media era. J. Beijing Inst. Print. Print. 1–6. doi: 10.19461/j.cnki.1004-8626.2022.03.004

[ref105] Wang Z. He Q. Blum R. S. (2021). Exploiting information about the structure of signals of opportunity for passive radar performance increase. IEEE Trans. Signal Process. 69, 6083–6100. doi: 10.1109/TSP.2021.3099627

[ref106] Weick K. E. Roberts K. H. (1993). Collective mind in organizations: heedful interrelating on flight decks. Adm. Sci. Q. 38, 357–381. doi: 10.2307/2393372

[ref107] West C. P. Dyrbye L. N. Rabatin J. T. Call T. G. Davidson J. H. Multari A.. (2014). Intervention to promote physician well-being, job satisfaction, and professionalism: a randomized clinical trial. JAMA Intern. Med. 174, 527–533. doi: 10.1001/jamainternmed.2013.14387, PMID: 24515493

[ref108] Wolever R. Q. Bobinet K. J. McCabe K. Mackenzie E. R. Fekete E. Kusnick C. A.. (2012). Effective and viable mind-body stress reduction in the workplace: a randomized controlled trial. J. Occup. Health Psychol. 17:246. doi: 10.1037/a0027278, PMID: 22352291

[ref109] Wu M. L. (2009). Structural equation Modeling: Operation and application of AMOS. Chongqing, China: Chongqing University Press.

[ref110] Wu C. X. (2016). A brief analysis of the professional background, characteristics and development paths of internet editors. Chin. Edit. 5

[ref111] Xanthopoulou D. Bakker A. B. Demerouti E. Schaufeli W. B. (2007). The role of personal resources in the job demands-resources model. Int. J. Stress. Manag. 14, 121–141. doi: 10.1037/1072-5245.14.2.121, PMID: 36385035

[ref112] Xanthopoulou D. Bakker A. B. Demerouti E. Schaufeli W. B. (2009). Reciprocal relationships between job resources, personal resources, and work engagement. J. Vocat. Behav. 74, 235–244. doi: 10.1016/j.jvb.2008.11.003, PMID: 32619287

[ref113] Xiong H. X. Zhang J. Ye B. J. Zheng X. Sun P. Z. (2012). Common method variance effects and the models of statistical approaches for controlling it. Adv. Psychol. Sci. 20:757. doi: 10.3724/SP.J.1042.2012.00757

[ref114] Xu Y. Y. Lin X. Q. (2021). How does workplace loneliness affect employee performance?—based on the perspective of job requirements and resource theory. Econ. Manag. 6, 69–83. doi: 10.19616/j.cnki.bmj.2021.06.005

[ref115] Zaphiris P. Ioannou A. (Eds.). (2016). Learning and collaboration technologies: third international conference, LCT 2016, held as part of HCI International 2016, Toronto, ON, Canada, July 17–22, 2016, proceedings (Vol. 9753). Springer.

